# Protocol for a randomized controlled trial of early prophylactic feeding via gastrostomy versus standard care in high risk patients with head and neck cancer

**DOI:** 10.1186/1472-6955-13-17

**Published:** 2014-07-01

**Authors:** Teresa Brown, Merrilyn Banks, Brett Hughes, Lizbeth Kenny, Charles Lin, Judith Bauer

**Affiliations:** 1 Centre for Dietetics Research (C-DIET-R), School of Human Movement Studies, The University of Queensland, St Lucia, Brisbane, QLD 4072, Australia; 2 Department of Nutrition and Dietetics, Royal Brisbane and Women’s Hospital, Herston, Brisbane, QLD 4029, Australia; 3 Cancer Care Services, Royal Brisbane and Women’s Hospital, Herston, Brisbane, QLD 4029, Australia

**Keywords:** Prophylactic, Gastrostomy, Head and neck cancer, Nutrition support, Enteral nutrition, Quality of life

## Abstract

**Background:**

Patients with head and neck cancer are at high risk of malnutrition and dysphagia. Enteral tube feeding via a gastrostomy or nasogastric tube is often required in response to dysphagia, odynophagia or side effects of treatment that lead to dehydration and/or weight-loss. A recent systematic review concluded that the optimal method of tube feeding remains unclear; however prophylactic gastrostomy, placed in anticipation of its use during and after treatment, is common practice, following a number of demonstrated benefits. However the majority of these studies have been undertaken in patients receiving radiotherapy alone. More recent studies in patient populations receiving concurrent chemoradiotherapy are showing that despite prophylactic gastrostomy placement significant weight loss still occurs, placing the patient at risk of the consequences of malnutrition. Therefore we set out to investigate innovative prophylactic nutrition support via the gastrostomy to optimise the nutritional outcomes of patients with head and neck cancer.

**Methods/Design:**

Patients with head and neck cancer will be eligible for this single centre randomised controlled trial if they are identified for referral for a prophylactic gastrostomy using local guidelines. Patients will be excluded if they are: under the age of eighteen; pregnant; unable to give informed consent; or severely malnourished or moderately malnourished with significant dysphagia requiring a liquid or puree diet. All eligible patients who consent for the study will be allocated randomly to either the intervention or control group (usual care). The intervention group will commence prophylactic supplementary nutrition support via the gastrostomy immediately following placement compared to usual care where nutrition support is commenced via the gastrostomy when clinically indicated during treatment. Key outcome measures will be percentage weight loss, body composition, nutritional status and quality of life, measured at baseline and three months post treatment.

**Discussion:**

To our knowledge this is the first study to evaluate the effectiveness of early prophylactic tube feeding compared to commencement of feeding during treatment, as per current standard practice, in patients undergoing prophylactic gastrostomy prior to treatment for head and neck cancer.

**Trial registration:**

This trial has been registered in the Australian New Zealand Clinical Trials registry as ACTRN12612000579897.

## Background

Patients with head and neck cancer are at high risk of malnutrition and dysphagia. Evidence based guidelines are available to provide optimal nutrition care to this patient group throughout the treatment trajectory [1]. There is good evidence to support the role of dietetic counselling to improve nutrition outcomes in patients with head and neck cancer receiving radiotherapy [2]. Enteral tube feeding via a gastrostomy or nasogastric tube is often required in response to dysphagia, odynophagia or other side effects of treatment that lead to dehydration and/or weight-loss during or after cancer treatment. A recent systematic review concluded that the optimal method of tube feeding remains unclear [3], although this was based on only one eligible randomised controlled trial (RCT) comparing the use of nasogastric tubes and gastrostomy tubes placed when required during treatment [4]. Since then a new RCT has been published [5] which supports the use of gastrostomy over nasogastric tubes, however the actual timing of the tube placement in relation to the treatment is unclear. Two further RCTs have compared proactive to reactive enteral feeding approaches, and while less weight loss is seen with the proactive approach, the differences are not statistically significant [6,7] There are benefits and disadvantages to both types of enteral feeding and their timing [8].

Prophylactic placement of a gastrostomy, in anticipation of its use during and after treatment, is common practice. Consideration must be given to the risks associated with the procedure of gastrostomy placement [9] and there is increasing concern that gastrostomy placement leads to prolonged tube dependency and long term dysphagia [4,10,11]. Although one additional study comparing two different centre’s approaches with reactive gastrostomy placement versus proactive gastrostomy placement found a higher rate of gastrostomy dependency in the proactive placement group at three months there was no difference at six or 12 months [12]. The risks of gastrostomy placement are often outweighed by the number of benefits that have been demonstrated with a prophylactic gastrostomy tube with, earlier commencement of nutrition support [13], reduced weight loss [14,15], improved quality of life [6,16,17], and reduced admissions and healthcare costs [18-20]. Of note the majority of these studies have been undertaken in patients receiving radiotherapy alone. As concurrent chemoradiotherapy is the generally accepted non-surgical standard of care for many of these patients, the additional toxicities associated with this dual modality treatment place the patient at greater nutritional risk. More recent studies are showing that despite prophylactic gastrostomy placement significant weight loss still occurs [7,21,22], placing the patient at risk of the consequences of malnutrition. One large study of patients receiving chemoradiotherapy which compared reactive (n = 279) and proactive (n = 166) gastrostomy placement reported no difference in weight loss outcomes between the two groups but the proportion of patients with weight loss >10% at the end of treatment was 37-42%, and by 12 months post treatment is was 42-53% [12]. A separate study utilising a reactive feeding approach with nasogastric tubes also reported a mean weight loss of 10.4% during treatment, with a weight loss of >10% affecting over half of their patients (54%) [23]. Similar to the literature, our current clinical practice guidelines with prophylactic gastrostomy tubes [24], whilst effective in reducing unplanned hospital admissions and length of stay [20], did not appear to be effective in achieving positive nutrition outcomes [25]. We have hypothesised from clinical observation that this may be due to: poor tolerance to nutrition support during treatment due to the side effects; difficulties adhering to nutrition recommendations secondary to other barriers such as time, finance or fatigue; increased nutritional requirements during treatment; and/or reluctance of patients to engage with utilising the tube for nutrition support. Therefore it was hypothesised as to whether earlier nutrition intervention can improve these patient’s nutritional outcomes.

The majority of studies published in the literature utilising prophylactic gastrostomy tubes generally commence nutrition support when clinically indicated in response to deterioration in swallowing or nutritional status [13,26,27]. Some studies have reported on commencement of enteral feeds prior to treatment [28-31] however these study designs are quite different to our proposal, with their patients often having a poor nutritional status or dysphagia at baseline therefore requiring immediate or therapeutic nutrition support, in comparison to our target population being primarily well nourished with minimal dysphagia. Whilst this innovative pre-treatment nutrition intervention strategy of prophylactic feeding may not address all our concerns or barriers experienced with current practice with gastrostomy feeding, the intervention is intended to particularly aid the psychological aspects.

Anecdotally in practice, we have seen that patients who have a gastrostomy placed for surgery and then require nutrition support again during radiotherapy, appear to be better adapted to the tube, than patients who have had the tube placed for definitive chemoradiotherapy. This observation was supported by recent studies in the literature where Salas et al. reported a negative physical effect of the gastrostomy just after the beginning of treatment, but also a positive mental effect over time as patients became used to the tube [6]. Merrick et al. also recently explored patient experiences with a gastrostomy tube through a qualitative study design, and three themes were found [32]. The majority of patients demonstrated “constructive cognitive appraisal” whereby they positively adapted to the tube and accepted tube feeding. However some patients were described as displaying “cognitive affective dissonance”, whereby although they experienced no physical concerns with the tube, there was a negative mental reaction to the tube. Smaller numbers of patients were found to have “emotion focused appraisal” so while accepting the necessity of the tube, there was a lot of anxiety and fear. Therefore part of the rationale for this current study design is to also assist patients with adapting to their tube by commencement of earlier tube feeding, so that when tube feeding becomes absolutely necessary during treatment, there is less anxiety and it becomes easier to adapt to and manage.

This study aims to compare the outcomes of early prophylactic tube feeding compared to commencement of feeding during treatment, as per current standard practice, in patients undergoing prophylactic gastrostomy prior to treatment for head and neck cancer. The research will directly inform future clinical practice and will provide more evidence on the role of pre treatment nutrition support in this patient population.

## Methods/Design

### Study design and setting

This study is a single centre randomised controlled trial. Recruitment will be from patients attending the Combined Head and Neck Clinic at the Royal Brisbane and Women’s Hospital, a tertiary hospital for cancer care services in Queensland, Australia. The study has been approved by the Royal Brisbane and Women’s Hospital Human Research Ethics Committee on 19th July 2012 (HREC/12/QRBW/162) and The University of Queensland Medical Research Ethics Committee on 8th August 2012 (2012000890).

### Participants and recruitment

Patients will be eligible for the study if they are identified as high risk patients and referred for a prophylactic gastrostomy as per the Royal Brisbane and Women’s Hospital Swallowing and Nutrition Management Guidelines for Patients with Head and Neck Cancer [24]. A patient would be deemed high risk at the multidisciplinary combined head and neck clinic if they meet the following criteria: oral cavity or oropharyngeal cancer receiving bilateral chemoradiotherapy; nasopharyngeal or hypopharyngeal cancer receiving chemoradiotherapy; or an unknown primary tumour receiving chemoradiotherapy; or if they have severe malnutrition. The final decision of tube placement is made by the treating team. Patients will be excluded if they: are under the age of eighteen; are pregnant; have cognitive impairment, an intellectual disability or a mental illness; or are receiving non-curative intent treatment. Patients meeting these criteria will be identified by a member of the treating team (radiation oncologist, medical oncologist or surgeon) who will discuss the study and provide a copy of the Patient Information and Consent Form if they are interested. The research dietitian (author TB) will be notified of these patients and a follow up telephone call will be made to provide full details of the study and to answer any patient questions. If the patient wishes to enroll the research dietitian will assess the patient when they attend for their gastrostomy procedure to complete eligibility criteria. A full nutritional assessment will be undertaken using the Patient Generated Subjective Global Assessment (PG-SGA) tool [33]. This is a validated tool recommended to assess nutritional status in patients with cancer [34], and encompasses assessment of weight history, dietary intake, nutrition impact symptoms, function/activity, and physical assessment of muscle/fat stores. The tool provides a subjective global assessment (SGA) rating of well nourished (SGA A), moderate malnutrition (SGA B) or severe malnutrition (SGA C). A numerical score is also generated indicating the level of nutritional risk. A score >9 indicates a referral to the dietitian for management. Patients will be excluded if they are found to have either: severe malnutrition or moderate malnutrition with significant dysphagia requiring a liquid or puree texture modified diet. Remaining eligible patients will provide written consent and baseline data will be collected.

### Interventions

All eligible patients who consent for the study will be allocated randomly to either the intervention or control group (usual care). All patients will receive education from the dietitian and nursing staff on the care of their gastrostomy tube pre treatment and will receive joint dietetic and speech pathology review on a weekly basis during treatment.

In the hospital’s usual care, patients will commence enteral nutrition via their prophylactic gastrostomy when required during treatment following assessment by the clinical dietitian. Indicators for commencing enteral nutrition are: oral intake less than 60% of estimated energy requirements (based on 125-145 kJ/kg) for a period of or anticipated to be, greater than 10 days; or the patient is unable to maintain weight; or the patient requires significant texture modification of diet; or the patient has increased or uncontrolled nutrition impact symptoms. The regimen will be determined by the clinical dietitian to suit the patients’ individual requirements and adapted as required during treatment. All patients are encouraged to maintain oral intake as much as possible during treatment and as long as it remains safe to do so as advised by the speech pathologist.

The intervention group will commence prophylactic nutritional support via their gastrostomy at the time of tube placement (pre treatment) in addition to their current oral intake. The supplementary enteral nutrition will consist of a 200 ml bolus feed (1.5 kcal/ml polymeric formula) to be administered twice a day between meal times, and will continue until completion of treatment. The enteral nutrition products for this intervention will be provided to the patient by the Department of Nutrition and Dietetics. Adherence will be monitored by the patient completing a self-reporting diary and verified by the clinical dietitian. If during treatment the patient displays any of the clinical indicators described above in usual care, the enteral feeding regimen will be increased by the clinical dietitian to suit the patients’ individual requirements.

All patients will be referred to their local dietitian service on completion of treatment for ongoing care as required. The research dietitian will provide monthly telephone review with all patients to determine ongoing use of the gastrostomy tube for a maximum of six months post treatment.

### Objectives

The aim of this study in patients undergoing prophylactic gastrostomy prior to treatment for head and neck cancer is to compare the nutritional, clinical and patient outcomes following an early prophylactic tube feeding approach versus our standard care of commencing tube feeding via the prophylactic gastrostomy as required. It is anticipated that the intervention group will have improved nutritional outcomes, which in turn is expected to improve patient outcomes, such as quality of life, and clinical outcomes, due to the earlier commencement of nutritional support. The null hypothesis is that there is no difference in the outcomes between the two groups.

### Outcomes

The primary endpoint is percentage weight change from baseline (pre treatment) to 3 months post treatment. Secondary endpoints will also be measured at baseline and 3 months post completion of treatment and include: nutritional status using the Patient-Generated Subjective Global Assessment tool (PG-SGA), body composition using Bioelectrical Impedance Analysis (BIA), and quality of life using the European Organisation for Research and Treatment of Cancer (EORTC) tools. The questionnaires to be used include the quality of life of cancer patients (EORTC QLQ-C30) and the module for head and neck (EORTC QLQ-H&N35). Additional qualitative data will be collected through the patient diary to assess adherence to the intervention. If patients are unable to meet the recommended goal intake, the reason why will be recorded by the patient e.g. nausea, lack of time, too tired etc. Tertiary endpoints will include: tolerance to chemotherapy and radiotherapy (dose received, changes to treatment, interruptions); unplanned hospital admissions; gastrostomy complications; and treatment response. Follow up to assess gastrostomy use (duration and degree of tube use post treatment) will occur monthly for six months post treatment or until tube is removed. If the tube is still in situ at six months, then follow up would also occur at 12 months post treatment to assess long term gastrostomy dependency. Survival will also be measured at 1 years and 5 years. The primary and secondary outcomes have been set at three months to ensure maximum follow up and prevent attrition from the study. As the centre is a major tertiary hospital in Queensland which covers an area of 715 309 square miles, many patients do not receive ongoing follow up at the tertiary centre, but at regional cancer centres or local health services. In addition the aim of this study is to see whether an early nutrition intervention can minimise weight loss in the acute period of treatment. Other studies have shown the nadir of weight loss occurs around this time point [25,35], and looking at weight outcomes beyond this time point can be impacted upon by so many other variables such as the effect of recurrent disease or a change in the nutrition management goals from weight maintenance during treatment to achieving a healthy weight through lifestyle and dietary changes as recommended for cancer survivorship. Therefore the aim of this study was to investigate the effect of an early nutrition intervention on minimising this nadir of weight loss.

To enhance the quality of the measurements and to reduce any inter-rater variability in assessment the data will be collected by the designated research dietitian. The research dietitian is responsible for recruitment and data collection only, and is not providing any dietetic patient care.

### Adverse events

A data monitoring committee will meet quarterly to review and collate serious adverse events. Any serious adverse events resulting from the study intervention will be reported to the RBWH Human Research and Ethical Committee as per standard procedures, and would be appropriately managed by the medical team to minimise or eliminate the effect of the adverse event on the patient.

### Intervention team roles

The study intervention team consists of: one research dietitian (author TB) who is responsible for the recruitment and outcome assessments at baseline and follow-up, and a second research dietitian (author MB) who is responsible for the randomisation process. The dietetic care is provided by two different treating clinicians (not blinded): one is allocated to the care of the standard arm; the second is allocated to the care of the intervention arm. This is to prevent any cross contamination between the cares given in each arm of the study. The multidisciplinary treatment team is also notified of the randomisation outcome so that there is reinforcement of the nutrition protocol from all professions involved in the patient care. The research dietitians are not involved in the clinical care of the patient. The research dietitian responsible for recruitment and outcome assessment is blinded to the randomisation sequence but not to the randomisation outcome, as they also provide support and encouragement to the intervention group to maximise adherence to the protocol.

### Sample size

The primary outcome in this study is a continuous response variable (% weight loss) from independent control and intervention patients, which have been randomised in a ratio 1:1. In a previous study we have found high risk patients recommended for a prophylactic gastrostomy (i.e. the target population for this RCT) to experience a mean 9% weight loss at three months post treatment with standard deviation 9.4 [25]. The aim of this intervention is to reduce this weight loss; however there are no other studies which have investigated the impact of prophylactic enteral tube feeding on which we can draw an anticipated response for this study for the sample size calculation. Ideally the goal for the intervention group is a mean weight loss of <5% as this is not deemed to be clinically significant, and thus a positive nutrition outcome. Therefore to detect a 5% true difference in% weight loss between the intervention and control groups, 56 patients in each group will be required to be able to reject the null hypothesis that the population means of the intervention and control groups are equal with probability (power) 0.8. The Type I error probability associated with this test of this null hypothesis is 0.05. Given that mortality during treatment is low and good follow up at three months is expected, as patients are returning to the hospital for their treatment scans and appointment with the oncologist, an attrition rate of 10% is used. Therefore the total sample required is 123 patients (62 patients in each group).

There is a study to consider of patients receiving chemoradiotherapy in which an early pre-treatment nutrition intervention of dietary counselling with or without oral supplements or tube feeding was compared to patients without a pre treatment nutrition assessment and intervention. They found a mean weight loss at three months post treatment of 9.75% +/− 5.75 in their standard care group compared to a mean weight loss of 2.77% +/− 7.93 [35]. Therefore the sample size was also calculated based on this demonstrated difference of 7% and examples of multiple sample sizes based on varying assumptions are illustrated in Table 1. Therefore sample size could be reduced to 39 patients per arm, with an attrition rate of 10%, which then results in total sample size of 86 (43 patients in each group), at a power of 90%.

**Table 1:** Examples of sample sizes required with varying assumptions and a Type I Error of 0.05

	**Number of patients required in each arm**			
**Power**	**SD = 9.4**[25]		**SD = 5.34**[35]	
	5% difference*	7% difference**	5% difference*	7% difference**
**80%**	**56**	**29**	**19**	**10**
**90%**	**75**	**39**	**25**	**13**

*Difference to reach clinically significant target weight loss of <5% (as studies have shown weight loss of 9.3-9.75%) [25,35].

**Difference seen with pre-treatment dietary counselling intervention [35].

Both of these sample sizes are deemed feasible from historical data at our institute. Each year approximately 500–550 patients attend the Combined Head and Neck Clinic at our institution. In a sample year (2010–11) 270 patients went on to have curative treatment with a standard referral to the dietitian. Of these; 106 patients were planned for adjuvant or definitive concurrent chemoradiotherapy with 88 categorised as high risk. The majority of these proceeded with a prophylactic gastrostomy as per the guideline recommendation (n = 86), and nil were considered high risk due to severe malnutrition. Based on 86 eligible patients per year, factors such as patients not giving consent (20%) and other factors such as scheduling difficulties (5%) were considered, resulting in an estimated 64–65 patients per year (or approximately 5 patients per month). Therefore recruitment is anticipated to be achievable in 2 years from a single centre.

### Randomisation

The research dietitian (author TB) will inform the clinical treating dietitian of newly recruited patients following consent and baseline data collection. The clinical dietitian will provide patient details to an independent member of the research team (author MB) for randomisation. The random allocation sequence is generated by computer and stratified according to baseline nutritional status (well nourished versus moderate or suspected malnutrition). Additional stratification based on tumour site or treatment was not undertaken, as the high risk category of the guidelines used to determine prophylactic gastrostomy placement has been based on these factors. As the guidelines have been found to be highly specific at predicting the requirement for a gastrostomy [24] it was not deemed necessary that additional stratification was required. It is not possible for this study to be double-blinded; however the allocation sequence is concealed to the participants, their health care providers, and the research dietitian responsible for recruitment, as it is undertaken by an independent member of the research team not involved in patient care or data collection.

### Statistical methods

To determine whether there are any differences between the two groups at baseline - participant characteristics will be summarised using mean and standard deviation for continuous variables and analysed using student t-tests, and categorical variables will be summarised using frequencies and percentages and analysed using chi-squared tests of association. If the data is not normally distributed and found to be skewed, then the data can be transformed using log transformation before applying the appropriate parametric test. If this is not successful, then alternative non parametric tests can be applied, such as the Wilcoxon-Mann–Whitney test for continuous variables.

Analysis will be undertaken on an intention to treat basis. To determine the effect of the nutrition intervention group versus the control group (the independent categorical variable) on the primary outcome of % weight loss (the dependent continuous variable), analysis of variance (ANOVA) will be used. For the secondary outcomes,% fat free mass, PG-SGA score and quality of life score, the change over time from baseline to 3 months will be analysed between groups using the repeated measures ANOVA. Univariate analysis will be used to determine the association of any other independent variables on the outcome measures. Any statistically significant associations found will then be used to add each variable into a normal regression model along with any other clinically important variables such as the tumour site, staging, treatment type, and baseline BMI and weight loss. Age and gender will also be added to adjust for any confounding effects. The regression model will also enable adjusting for any important clinical differences found between the intervention and control group at baseline.

All tests will assume normal distribution; otherwise alternative non parametric tests will be applied for non-normal distributed data. Statistical significance will be set at p < 0.05. All outcomes will be reported in the future report.

## Discussion

This study will evaluate the effectiveness of prophylactic nutrition support via a gastrostomy compared to current standard practices of commencing nutrition support in a reactive way during treatment. To our knowledge this is the first study to trial this approach of prophylactic nutrition support in a formal research setting. Of those studies that report specific details on the actual timing of the commencement of nutrition support, all commence a number of days after the commencement of treatment: 1–33 days [26], 21 days [27] and 10 days [13]. See Table 2. The studies which have reported on the early commencement of tube feeding have quite a different study design and intent to our protocol. See Table 3. Nguyen et al. stated criteria in their study for the initiation of enteral feeds prior to treatment being; poor oral intake prior to treatment, or aspiration on pre treatment Modified Barium Swallow assessment [28]. However the authors did not describe the number of patients in their study that met these criteria. Wiggenraad et al. reported 26% of their patients commenced enteral feeds prior to treatment [29], however this was likely in response to poor swallowing or nutritional status, with 48% of patients already requiring a puree or liquid diet and 78% of patients with weight loss prior to treatment. It was also found that when there was dysphagia at baseline, tube feeding was commenced earlier (day 2) compared to when there was no dysphagia at baseline (day 17). There have been two other studies which have looked at initiating tube feeding immediately following prophylactic placement [30,31]. Both of these studies also had a significant proportion of patients who were nutritionally compromised at baseline and therefore the characteristics of the patients commencing early nutrition support are likely to be quite different to the patient characteristics we are anticipating studying. Both also appear to provide the majority of nutrition via the gastrostomy tube, with minimal oral intake. This is a very different approach to our proposed intervention where we are providing supplementary nutrition via the tube and advocate patients maintain/continue with oral intake for as long as possible to minimise the risk of long term dysphagia and prolonged rehabilitation post treatment [36]. Salas et al. reports on a randomised controlled trial on the quality of life in patients with a systematic gastrostomy (n = 21) versus no systematic gastrostomy (n = 18) [6]. In the control group 13 patients later receive an insertion of a gastrostomy and the other 5 patients remained on oral intake alone. There are no details in the methodology on the nutrition interventions provided to either group and the results do not include information on the actual timing of gastrostomy placement or the timing for the commencement of feeding. Therefore it is difficult to ascertain whether prophylactic nutrition support was utilized or whether it was prophylactic (systematic) tube placement.

**Table 2:** Comparison of key studies which report on timing of commencement of enteral feeding during treatment

**Citation**	**Study population**	**N**	**Type and timing of tube placement**	**Commencement of feeds (no. days after start of treatment)**	**Outcomes**
Nugent et al., *Journal of Human Nutrition and Dietetics* 2010, 23(3):277-284	HNC treated with radical chemoradiotherapy requiring tube feeding	50	Prophylactic PEG (before start of treatment) n = 21	Range: 1–33 days	Mean weight loss: −4.6%
			Late PEG (during treatment) n = 11	Range: 14–30 days	Mean weight loss: −8.7%
			NG tube n = 18	Range: 10–34 days	Mean weight loss: −8.5%
Raykher et al., *JPEN* 2009, 33(4):404-410	HNC with definitive or adjuvant chemoradiotherapy or radiotherapy requiring PEG	163	Prophylactic PEG (before start of treatment) n = 161	Mean 21 days	PEG used by n = 160 (98%) due to severe dysphagia (mean duration of use 251 +/− 317 days)
			Late PEG (during treatment) n = 2		
					Treatment interruptions in 7%
					Strictures requiring dilatation in 12%
					BMI optimised in obese/overweight patients through individual regimens
Scolapio et al., *Journal of Clinical Gastroenterology* 2001, 33(3):215-217	HNC with definitive or adjuvant radiotherapy requiring PEG	54	Prophylactic PEG (before start of treatment) n = 41	Mean 10 days	Mean wt loss 2.7 kg
					Nil nutrition related admissions
			Late PEG (during treatment) n = 13	Mean 23 days	Mean wt loss 4.5 kg
					Nutrition related admissions n = 4

KEY: HNC = Head and Neck Cancer; PEG = percutaneous endoscopic gastrostomy; NG = nasogastric tube; BMI = body mass index.

**Table 3:** Comparison of key studies which commence enteral feeding via a prophylactic gastrostomy immediately

**Citation**	**Study population (N)**	**Study design**	**Nutrition characteristics at baseline**	**Timing of PEG tube placement**	**Commencement of PEG feeds**	**Oral intake**	**Outcomes**
Marcy et al. *Supportive Care in Cancer* 2000, 8(5):410-413	Stage IV HNC treated with chemoradiotherapy and prophylactic PEG (n = 50)	Retrospective case series	34% had BMI <20 kg/m2	Within 5 days before treatment (n = 38) or within 5 days after treatment started (n = 12)	All patients started 48 hours post insertion	Unknown	Mean weight increase of 2.5 kg by 3 weeks
					Tube feeds were increased over 4 days to provide goal of 2000 kcal/day		
Beer et al. *Nutrition and cancer* 2005, 52(1):29-34	HNC with radical radiotherapy or radiochemotherapy and PEG tube feeding (n = 151)	Retrospective comparative cohort	Group A – 49% malnourished	Group A (n = 78, 52%) early PEG: before or within 2 wk of radiotherapy.	All patients started 12 hours post insertion	Clear fluids only	Mean weight loss was 1.03 kg in group A vs. 4.0 kg in group B, (P = 0.004)
			Group B – 47% malnourished	Group B (n = 73, 48%) delayed PEG: after 2 wk of radiotherapy.	Tube feeds were increased over 3 days to provide individual goal	Clear fluids only	Treatment interruptions of >3 days was 10% in Group A vs. 25% in Group B (P = 0.02)
Wiggenraad et al., *Clinical otolaryngology* 2007, 32(5):384-390	Stage III and IV HNC treated with chemoradiotherapy and prophylactic PEG (n = 50)	Retrospective case series	48% on puree or liquid diet	Mostly 1–2 weeks before treatment commenced (n = 3 had placed >3 weeks prior)	26% commenced prior to treatment (tube feeding initiated if reduced food-intake or weight loss)	Unknown	Mean loss of weight during treatment 2.8%
			78% had weight loss				

KEY: HNC = Head and Neck Cancer; PEG = percutaneous endoscopic gastrostomy; BMI = body mass index.

Some of the anticipated problems with this current study protocol will be patient adherence to recommendations. Capuano et al. have shown that patients who adhere to dietary recommendations have improved outcomes compared to those that do not adhere [37]. Therefore patients have been asked to keep a record of the number of feeds they have each day so that adherence can be considered as a confounding variable. Patients have also been asked to record any reasons why they have not had the recommended amount. This may be due to feeling full or bloating, or from side effects of treatment such as nausea, or from practical issues such as not having enough time in the day due to treatment schedules/appointments, being fatigued or financial difficulties to purchase the recommended feeds. This additional information from the study will provide evidence for the feasibility of implementing the early intervention into clinical practice, and will also assist in identifying factors that patients experience during treatment so that alternative strategies can be found.

With the advent of evolving techniques using intensity modulated radiotherapy and helical tomotherapy, the dose to surrounding tissues (pharyngeal constrictor muscles) and salivary glands can be spared due to steeper dose gradient outside target volumes, and as a consequence there are less reports of long term dysphagia and xerostomia [38-40]. Therefore it is hypothesized that patients may be less likely to require feeding tubes. The guidelines for prophylactic gastrostomy at our institution were previously validated in a patient cohort receiving standard 3D conformal radiotherapy [24] and so additional research is also planned to retrospectively re-validate these guidelines in patients now receiving helical tomotherapy to see whether the requirement for a feeding tube has changed. However it should be noted there are a range of other reasons why patients may have an inadequate oral intake other than dysphagia and xerostomia. Therefore these patients are still susceptible to weight loss and malnutrition, and the adverse consequences. So despite the reduction of some side effects in this population with evolving treatments, nutrition support can still be indicated for other reasons. Patients in this study protocol are being asked to record the main reason they are using the gastrostomy for nutrition support e.g. pain on swallowing (odynophagia), difficulty swallowing (dysphagia), painful ulcers (mucositis), difficulty chewing/dry mouth (xerostomia), taste changes (dysgeusia), nausea/vomiting or general poor appetite. These nutrition impact symptoms can all lead to a decline in nutritional status and weight loss, both of which have been associated with poor quality of life [41,42].

In conclusion the results from this study will aim to address the questions regarding the optimal nutrition interventions required to manage patients with head and neck cancer and will also assist in our understanding of the impact of the new treatment regimens. Together this will inform future clinical practice for the nutritional management of this patient population to achieve positive outcomes.

## Abbreviations

RCT: Randomised controlled trial; PG-SGA: Patient generated - subjective global assessment; BIA: Bioelectrical impedance analysis; EORTC: European Organisation for Research and Treatment of Cancer; BMI: Body mass index.

## Competing interests

None of the authors have any competing interests relevant to this trial to disclose.

## Authors’ contributions

TB is a PhD student and conceived the study, contributed to the design of the study, and is responsible for patient recruitment, data collection, data analysis and drafting of the manuscript. MB, BH, LK, CL and JB contributed to the study design, data analysis interpretation and critical revision of the manuscript for important intellectual content. All authors read and approved the final manuscript.

## Pre-publication history

The pre-publication history for this paper can be accessed here:

http://www.biomedcentral.com/1472-6955/13/17/prepub
